# Maternal n-7 Unsaturated Fatty Acids Protect the Fetal Brain from Neuronal Degeneration in an Intrauterine Hyperglycemic Animal Model

**DOI:** 10.3390/nu15153434

**Published:** 2023-08-03

**Authors:** Haruka Okami, Ritsuko Kawaharada, Hitomi Yoshizaki, Akiyo Toriumi, Saki Tsutsumi, Akio Nakamura

**Affiliations:** 1 Department of General Surgical Science, Graduate School of Medicine, Gunma University, Maebashi 371-8511, Japan; m2220010@gunma-u.ac.jp; 2 Department of Health and Nutrition, Takasaki University of Health and Welfare, Takasaki 370-0033, Japan; nasu@takasaki-u.ac.jp; 3 Department of Bioregulatory Science (Physiology), Nippon Medical School, Tokyo 113-8602, Japan; hitomi-yoshizaki@nms.ac.jp; 4 Department of Public Health, Graduate School of Medicine, Gunma University, Maebashi 371-8511, Japan; m2320028@gunma-u.ac.jp; 5 Department of Neurophysiology & Neural Repair, Graduate School of Medicine, Gunma University, Maebashi 371-8511, Japan; m2310007@gunma-u.ac.jp; 6 Department of Molecular Nutrition, Faculty of Human Life Sciences, Jissen Women’s University, Hino 191-8510, Japan

**Keywords:** intrauterine hyperglycemia, glycation, apoptosis, n-7 unsaturated fatty acid

## Abstract

We previously reported that glycation induces insulin resistance in the hearts of newborn pups from a gestational diabetes mellitus (GDM) rat model. Administration of n-3 unsaturated fatty acids suppressed glycation and improved signaling in GDM rat pups. In this study, we investigated their effects on cranial neurons using the GDM rat model and PC12 cells derived from rat adrenal pheochromocytomas. Additionally, we examined whether n-3 and n-7 unsaturated fatty acids (*cis*-palmitoleic acid [CPA] and *trans*-palmitoleic acid [TPA]) ameliorate the detrimental effects of high glucose exposure on rats. In the neonatal cerebrum of GDM rats, increased levels of advanced glycation end products (AGEs) inhibited Akt phosphorylation; however, CPA and TPA intake during pregnancy ameliorated these abnormalities. Furthermore, exposure to high-glucose-induced apoptosis in PC12 cells compared to the cells cultured in control glucose. PC12 cells exposed to high-glucose with fatty acids exhibited reduced AGE production and apoptosis induction compared to the high-glucose group. These findings suggest that a hyperglycemic environment during pregnancy promotes AGE formation in brain neuronal proteins and induces apoptosis. Both TPA and CPA mitigated these abnormalities; however, CPA is cytotoxic, highlighting its safety in pregnant women.

## 1. Introduction

The nutritional status of mothers during pregnancy affects their long-term health. Barker et al. reported that low birth weight is associated with impaired glucose tolerance and cardiovascular diseases [1,2]; they proposed the Barker hypothesis, which states that prenatal undernutrition increases the risk of lifestyle-related diseases in adulthood [3]. Subsequently, Gluckman and Hanson proposed the Developmental Origins of Health and Disease (DOHaD) hypothesis, which states that a predisposition to lifestyle-related diseases is shaped by genetic and environmental interactions during fertilization, embryonic development, fetal life, and infancy and that over-nutrition after birth leads to the development of diabetes and hypertension [4,5]. The DOHaD hypothesis is supported by numerous epidemiologic studies that have shown that children born to pregnant women who are undernourished during pregnancy are at higher risk of developing several conditions, including coronary heart disease, hypertension, stroke, diabetes, obesity, and metabolic syndrome. For example, children born to undernourished mothers during the famine in the Netherlands during World War II were more likely than adults to develop lifestyle-related conditions such as glucose intolerance, lipid abnormalities, and ischemic heart disease [6,7]. Subsequently, many epidemiological studies have revealed that children born to undernourished pregnant women are at an increased risk of ischemic heart disease, stroke, hypertension, type 2 diabetes, chronic kidney disease, osteoporosis, malignancy, and psychiatric disorders [8,9,10,11]. To date, many DOHaD studies have reported the risks of nutritional deficiencies during pregnancy on the child’s health. However, only a few studies have investigated the impact of maternal overnutrition during pregnancy on the child’s health.

In recent years, the number of pregnant women with gestational diabetes mellitus (GDM) and type 2 diabetes mellitus has been increasing due to the westernization of diets and the increase in childbearing age. GDM is a common pregnancy complication with a global prevalence of 1.7–11.6% [12,13,14]. In 2010, the International Society for Diabetes and Pregnancy revised the diagnostic criteria for GDM based on a large observational study of approximately 25,000 pregnant women with impaired glucose tolerance conducted at 15 centers in 9 countries [15]. The study found that perinatal complications were significantly associated with maternal blood glucose levels even after adjusting for confounders such as maternal obesity [16]. In addition, infants born to pregnant women with impaired glucose tolerance during pregnancy develop complications in the neonatal period, such as congenital malformations, macrosomia, myocardial hypertrophy, and neonatal hypoglycemia [17,18,19]. After school age, a low incidence of gross and fine motor skills and a high incidence of neurological symptoms are indicative of attention-deficit/hyperactivity disorder [20,21,22]. These reports suggest the impact of nutrition on the future physical and mental activities of the child. However, the mechanisms by which maternal overnutrition leads to the development of psychiatric and nervous system diseases in children remain unclear.

We previously investigated cardiovascular complications in rat pups of GDM (PDM), established a rat model of GDM with intrauterine hyperglycemia during pregnancy, and examined the effects of PDMs on cultured hearts and primary myocardial cells [23]. A hyperglycemic environment during pregnancy causes excessive glycation of proteins (advanced glycation end products [AGEs]) in the heart and primary cultured myocardial cells of PDMs, resulting in inflammatory cytokine production and insulin resistance [23]. Furthermore, the administration of eicosapentaenoic acid (EPA), an n-3 unsaturated fatty acid that improves lipid metabolism, prevents thrombosis, and is used as a drug for cardiovascular disease, also improved the abnormalities caused by intrauterine hyperglycemia in the GDM rat model [23]. However, long-term intake of EPA by pregnant women increases the risk of maternal hemorrhage during childbirth [24,25] and possible mercury effects [26,27] owing to the high intake of EPA-rich fish.

In this study, we investigated the effects of palmitoleic acid, a functional lipid similar to EPA, in GDM model rats and cells exposed to a high-glucose medium. Palmitoleic acid is a 16-carbon monounsaturated fatty acid with cis-palmitoleic acid (C16:1 n-7 *cis*-type, CPA) and trans-palmitoleic acid (C16:1 n-7 *trans*-type, TPA) as structural isomers. CPA is abundant in nuts, such as macadamia nuts, and is biosynthesized from palmitic acid, a saturated fatty acid mainly found in the liver and adipose tissues [28]. TPA is produced by ruminant intestinal bacteria and is abundant in dairy products; it cannot be biosynthesized in the body and must be obtained via food [29]. Palmitoleic acid improves insulin sensitivity and inhibits cholesterol accumulation in humans [30,31,32]. Large observational and cross-sectional studies have revealed that TPA intake and plasma concentration are inversely correlated with insulin resistance and incidence of type 2 diabetes [33,34]. Only a few studies have compared and examined the molecular mechanisms underlying the formation of structural isomers of palmitoleic acid, CPA, and TPA.

Here, we used streptozotocin (STZ)-induced GDM rats to elucidate the effects of an intrauterine hyperglycemic environment on the neonatal cerebrum of rat pups. In addition, we analyzed the effects of CPA and TPA on palmitoleic acid levels in GDM rats to ameliorate the effects of the intrauterine hyperglycemic environment. Rat pheochromocytoma (PC12) cells cultured in a high-glucose medium were used to elucidate the molecular mechanisms associated with the effects of high glucose exposure. We explored the differences in the effects of its structural isomers, CPA and TPA, on PC12 cells with respect to palmitoleic acid, a candidate functional lipid that improves the effects of the intrauterine hyperglycemic environment.

## 2. Materials and Methods

### 2.1. Animals

All animal experiments were performed in accordance with the National Institutes of Health Guide for the Care and Use of Experimental Animals. The study protocols were approved by the Animal Committee of the Takasaki University of Health and Welfare (No. 2024; 37-1 Nakaorui-machi, Takasaki, Gunma 370-0033, Japan). Pregnant 15-week-old Wister rats (180–210 g weight) were purchased from CLEA Japan, Inc. (Tokyo, Japan) and divided into two groups: Control mother rat (*n* = 5) and GDM (*n*= 15) groups. GDM was experimentally induced in the GDM group via an intravenous injection of STZ (60 mg/kg; FUJIFILM Wako Pure Chemical Industries, Tokyo, Japan) in 50 mM citrate buffer (pH 4.5) on day 2 of gestation [35]. Pregnant rats with blood glucose levels > 350 mg/dl 24 h after STZ administration were assigned to the GDM group. Rats with GDM were administered CPA (*n* = 5, 150 mg/kg/day; Cayman Chemical, MI., USA), TPA (*n* = 5, 150 mg/kg/day; Larodan Fine Chemicals AB, Solna, Sweden), or water (*n* = 5) via a gastric tube daily from day 2 of gestation to delivery. The amounts of CPA and TPA used were the same as those used for diabetic rats [24]. The study comprised four groups; rat pups from control mother rats were designated (PCM, *n* = 10), while rat pups from GDM model rats administered CPA, TPA, and water were designated PDM/CPA (*n* = 10), PDM/TPA (*n* = 10), and PDM (*n* = 10), respectively. All the rats were fed a standard diet (CE-2; CLEA Japan Inc., Tokyo, Japan) throughout the gestation period. CE-2 diet contained 4.8% fat, 49.8% carbohydrate, 25.1% protein, 4.4% fiber, and 8.9% water, with a total caloric value of ~3424 kcal/kg (percent by weight). Animals were individually housed in plastic cages with woodchip bedding at a humidity of 60 ± 5% and subjected to 12 h light/dark cycle.

### 2.2. Cell Culture

Rat pheochromocytoma cells (PC12 cells; Riken Cell Bank, Tsukuba, Ibaraki, Japan) were cultured in the Roswell Park Memorial Institute (RPMI)-1640 medium (Thermo Fisher Scientific, Inc., Waltham, MA, USA) supplemented with 5% fetal bovine serum (FUJI-FILM Wako Pure Chemical Industries, Tokyo, Japan), 10% heat inactivated horse serum, 1% penicillin, streptomycin mixed solution, and 2 g/L sodium bicarbonate at 37 °C in a 5% CO_2_ humidified incubator. After seeding, PC12 cells were placed in the fresh RPMI-1640 medium containing 200 mg/dL glucose as the Control and 500 mg/dL glucose as the high glucose concentration, and incubated for seven days. To study the effects of unsaturated fatty acids on PC12 cells, 100 µM CPA (Cayman Chemical, MI, USA), 100 µM TPA (Larodan Fine Chemicals AB, Solna, Sweden), and 50 µM EPA (Tokyo Chemical Industry, Tokyo, Japan) were added to the high-glucose medium for seven days. The medium was changed every day. After seven days, neuronal differentiation was induced with 50 ng/mL of the nerve growth factor (NGF; FUJIFILM Wako Pure Chemical Industries, Tokyo, Japan) in RPMI-1640 medium supplemented with 1% FBS, 10% HS, and 10 mM HEPES (pH 7.0) after 2 days of culture [36].

### 2.3. Cell Viability Assay

Cell viability was examined using the Cell Counting Kit-8 (CCK-8; Dojindo, Kumamoto, Japan). PC12 cells (2 × 10^4^ cells/well) were seeded in 96-well plates and incubated for 24 h. Cells were treated with different fatty acid concentrations (50, 100, 200, and 300 µM) for 24, 48, and 72 h. Subsequently, 5 µL of CCK-8 solution was added to each well, and the cell were then incubated at 37 °C for 4 h. Absorbance was measured at 450 nm using a microplate reader (Corona Electric SH-1000; Hitachinaka, Ibaraki, Japan).

### 2.4. Immunoblotting Assay

On postnatal day 2, rat pups were anesthetized, killed with guillotine, and their whole brains were rapidly removed and rinsed with ice-cold 0.9% NaCl. The cerebrum was homogenized using the Tissue Protein Extraction Reagent (T-PER; Thermo Fisher Scientific Inc.) containing 5 mM EDTA, 1 × protease inhibitor cocktail (Millipore, Burlington, MA, USA), 1 × Phos STOP phosphatase inhibitors (Millipore) and 0.5 mM 4-(2- aminoethyl) benzenesulfonyl fluoride hydrochloride, and centrifuged at 10,000× *g* for 15 min at 4 °C. Total protein was separated using 12.5% sodium dodecyl sulfate-polyacrylamide gel electrophoresis (SDS-PAGE) and transferred to a polyvinylidene difluoride (PVDF) membrane (Clear blot P, ATTO, Japan). PC12 cells were homogenized using T-PER and centrifuged at 10,000× *g* for 15 min at 4 °C. Total proteins were separated by SDS-PAGE and transferred to a PVDF membrane.

Membranes were blocked with PVDF Blocking Reagent for Can Get Signal (Toyobo Co., Ltd., Osaka, Japan) or 3–5% bovine serum albumin (BSA) for 1 hour at 25 °C. Subsequently, the membranes were incubated with primary antibodies (1:1000) overnight at 4 °C, followed by secondary antibodies (1:5000) for 1 h at room temperature. Blots were visualized using the Immobilon Western Chemiluminescent HRP Substrate (Millipore) and quantitatively analysed using a Fusion S image analyzer (Vilber Lourmat, France).

Primary antibodies were anti-Akt (#9272), phospho-Akt (Ser-473) (p-Akt; #9267), caspase-3 (#14220), cleaved caspase-3 (#9664), SAPK/JNK (#9252), phospho-SAPK/JNK (p-JNK; #9251), p44/42 MAPK (Erk1/2; #9102), phospho-p44/42 MAPK (Erk1/2) (p-ERK; #9106), p38 MAPK (#9212), phospho-p38 MAPK (p-p38; #9216), Bcl-2 (#2764), Bcl-2-associated X (Bax; #2772), β-Tubulin (#2128), and β-Actin (#4970) were purchased from Cell Signaling Technology (Boston, MA, USA). AGEs (KH001) was purchased from Trans Genic Inc. (Fukuoka, Japan) and receptor of AGE (RAGE; ab3611) was purchased from Abcam (Cam-bridge, MA, USA). Anti-rabbit IgG, horseradish peroxidase (HRP)-linked antibody [#7074] and anti-mouse IgG, HRP-linked antibody [#7076] of secondary antibody were purchased from Cell Signaling.

### 2.5. Detection of Ubiquitinated Proteins

Total proteins were separated using SDS-PAGE and transferred to a PVDF membranes. Transferred membranes were blocked with 2% BSA for 1 h at room temperature, followed by hybridization with recombinant biotinylated human ubiquitin 1 tandem UBA (ubiquitin, 1: 1000; TUBE2-Biotin; R&D Systems, Inc., Minneapolis, MN, USA) for 1 h at room temperature. TUBE2-Biotin reactivity was detected using incubation with a biotinylated anti-Fc antibody and streptavidin–HRP conjugated antibody (1:5000; Tokyo Chemical Industry Co., Ltd., Tokyo, Japan). Protein bands were visualized using chemiluminescence as described above.

### 2.6. Measurement of Intracellular Reactive Oxygen Species (ROS) Levels

ROS levels were determined using the Cell ROX Green reagent (Thermo Fisher Scientific Inc.), according to the manufacturer’s protocol. PC12 cells seeded in an 18-well plate (3 × 10^4^/well) were incubated in a medium containing 5 µM CellROX Green reagent and PureBlu Hoechst 33342 Nuclear Staining Dye (Bio-Rad Laboratories, Hercules, CA, USA) at 37 °C for 30 min. The cells were washed three times with phosphate-buffered saline (PBS), and the medium was removed. Images were captured using a fluorescence microscope (BZ-X710; Keyence, Osaka, Japan). Absorbance with Cell ROX Green Reagent was measured at an excitation wavelength of 485 nm and emission wavelength of 520 nm, while that with Pure Blu Hoechst 33342 was measured at an excitation wavelength of 350 nm and emission wavelength of 461 nm.

### 2.7. Annexin V/Propidium Iodide (PI) Double Staining Assay

Cells were stained with annexin V-fluorescein isothiocyanate (FITC; MBL, Nagoya, Japan) and PI (Nacalai Tesque Inc., Kyoto, Japan) for 15 min. After drug treatment, PC12 cells were washed three times with PBS and mounted with 4’,6-diamidino-2-phenylindole (DAPI)-Fluoromount G (Southern Biotech, Birmingham, AL, USA). Stained cells were visualized under a fluorescence microscope (BZ-X710). Absorbance of annexin V was measured at an excitation wavelength of 494 nm and emission wavelength of 519 nm, PI at an excitation wavelength of 536 nm, and DAPI at an excitation wavelength of 358 nm and emission wavelength of 461 nm.

### 2.8. Statistical Analysis

Data are presented as the mean ± standard error. Statistically significant differences between experimental groups were analyzed using the Student’s *t*-test or analysis of variance, followed by multiple comparison Tukey’s test. Statistical significance was set at *p* < 0.05. Data were analyzed using the SPSS software version 25 (SPSS, Tokyo, Japan).

## 3. Results

### 3.1. Effect of a Hyperglycemic Environment and Functional Lipids during Pregnancy in GDM Rat Models

#### 3.1.1. Body Weights and Blood Glucose Levels of GDM Rat Pups

No differences in the mean body weights and blood glucose levels were observed among the rat pup groups (Table 1).

#### 3.1.2. AGEs, RAGE, and Insulin Signaling in GDM rat pups

**Figure 1:**
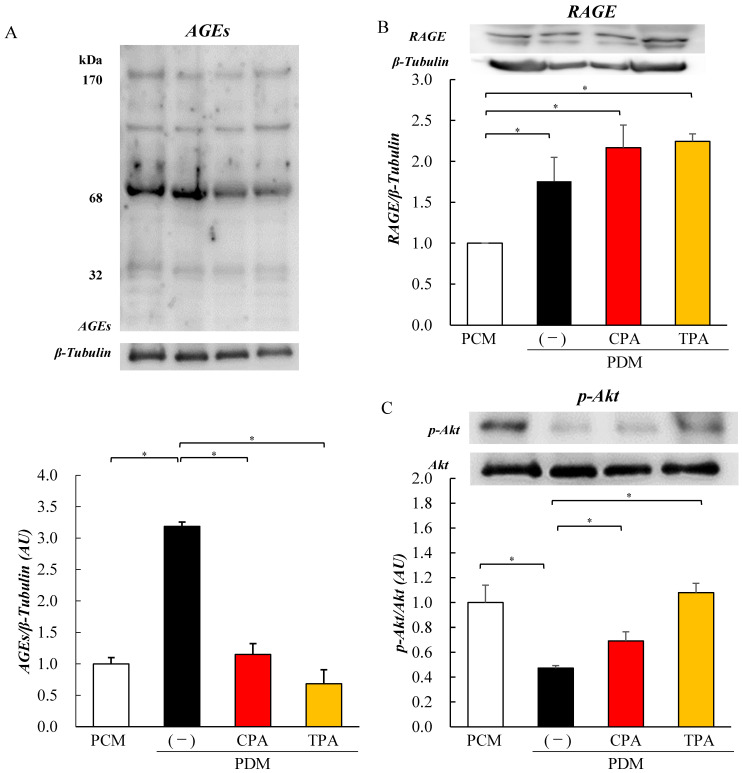
Effects of glucose on the levels of advanced glycation end products (AGEs), receptor of AGE (RAGE), and p-Akt in the neonatal brain (cerebrum). Brains (cerebrum) were obtained from the offspring of rat pups from control mother rats (PCM, *n* = 10) and rat pups of mothers with diabetes (PDM, PDM/CPA, and PDM/TPA, *n* = 10, respectively). Levels of AGEs (**A**). Levels of RAGE (**B**). Levels of p-Akt (**C**). * *p* < 0.05.

We analyzed the levels of AGEs, RAGE, and Akt phosphorylation in the neonatal brain (cerebrum) using Western blotting. The AGE level in the PDM group was higher than those in the PCM, PDM/CPA, and PDM/TPA groups (Figure 1A). However, the RAGE levels in the PDM, PDM/CPA, and PDM/TPA groups were higher than that in the PCM group (Figure 1B). The level of p-Akt was significantly lower in PDMs than in PCMs and higher in PDM/CPA and PDM/TPA than in the PDM (Figure 1C).

### 3.2. Effects of Unsaturated Fatty Acids on PC12 Cells Exposed to High-Glucose Medium

#### 3.2.1. AGEs, RAGE, and Insulin Signaling in PC12 Cells

**Figure 2:**
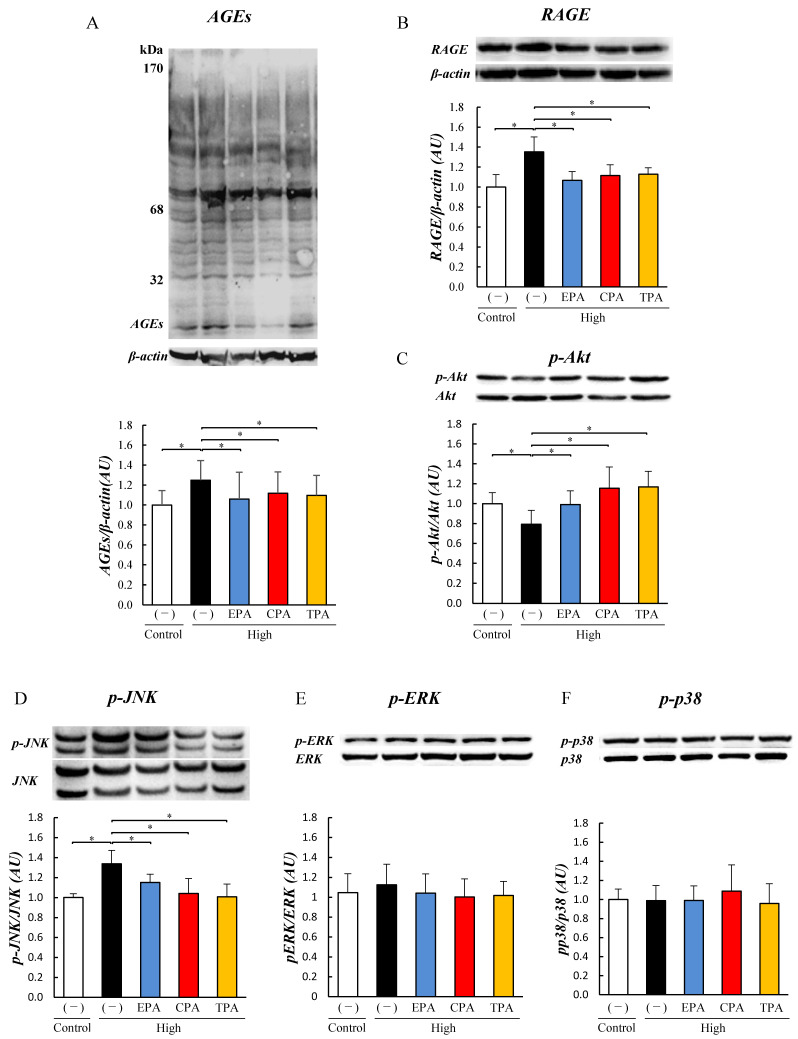
Effects of eicosapentaenoic acid (EPA), cis-palmitoleic acid (CPA), and trans-palmitoleic acid (TPA) on signaling in PC12 cells exposed to high glucose. Control PC12 cells and cells exposed to high glucose were prepared by culturing in 200 or 500 mg/dL glucose medium for seven days. PC12 cells were cultured in 500 mg/dL glucose medium supplemented with EPA, CPA, or TPA for seven days. The levels of AGEs, RAGE, p-Akt, p-JNK, p-ERK, and p-p38 in PC12 cells were measured using Western blotting. Generation level of AGEs (**A**). Level of RAGE expression (**B**). Levels of p-Akt (**C**), p-JNK (**D**), p-ERK (**E**), and p-p38 (**F**). Each value represents the average (±SE). * *p* < 0.05 vs. Control.

PC12 cells were exposed to a high-glucose medium to determine the effects of a high-glucose environment during pregnancy. In addition, the effect of unsaturated fatty acids on cells was examined by culturing PC12 cells in a high-glucose medium supplemented with unsaturated fatty acids (EPA, CPA, and TPA) for 7 days (High/EPA, High/CPA, and High/TPA). In the high medium, the generation levels of AGEs and the expression of RAGE were significantly increased compared to those in the control medium (Figure 2A,B). The generation of AGEs and the expression of RAGE were significantly suppressed in cells treated with EPA, CPA, and TPA compared with high (*p* < 0.05; Figure 2A,B).

Level of p-Akt was significantly lower in the high group than in the Control (*p* < 0.05; Figure 2C). When EPA, CPA, and TPA were added to the high-glucose medium, the level of p-Akt was significantly higher than that in the high-glucose medium (*p* < 0.05; Figure 2C). The level of p-JNK was increased in the high group compared to the Control but decreased in the high/EPA, high/CPA, and high/TPA groups (*p* < 0.05; Figure 2D), while the levels of p-ERK and p-p38 were not significantly different (Figure 2E,F).

#### 3.2.2. Apoptosis Signaling in PC12 Cells

**Figure 3:**
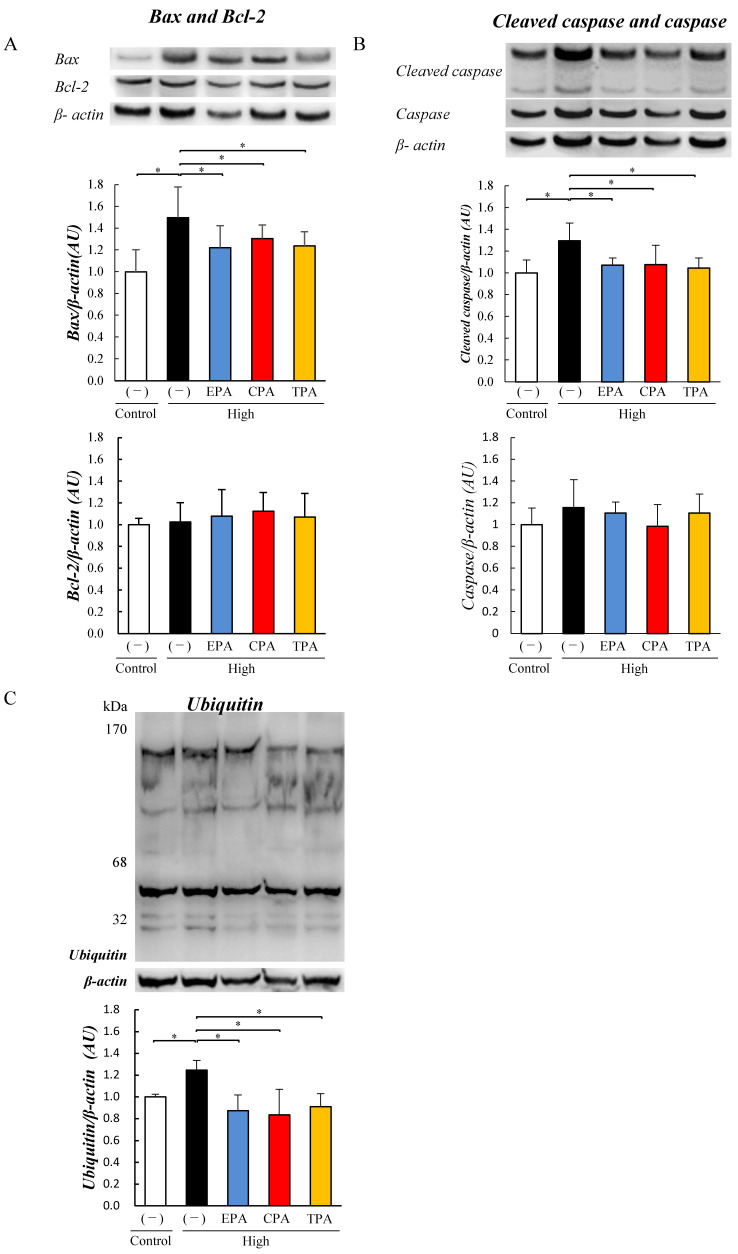
Effects of EPA, CPA, and TPA on apoptosis signaling expression in PC12 cells exposed to high glucose. Control and high groups were prepared by culturing PC12 cells in 200 or 500 mg/dL glucose medium for seven days. PC12 cells were cultured in 500 mg/dL glucose medium supplemented with EPA, CPA, or TPA for seven days. Bcl-2-associated X (Bax), Bcl-2, cleaved caspase, caspase, and ubiquitinated protein levels in PC12 cells were measured using Western blotting. Expression levels of Bax (**A**), Bcl-2 (**A**), cleaved caspase (**B**), caspase (**B**), and ubiquitinated protein (**C**). Each value is the average (±SE). * *p* < 0.05 vs. Control.

We examined the effects of EPA, CPA, and TPA on the apoptosis of PC12 cells exposed to high glucose. At high glucose levels, the levels of Bax and cleaved caspases in PC12 cells exposed to high glucose (high) were elevated compared to those in the Control (*p* < 0.05; Figure 3A,B). At high glucose levels, Bcl-2 and caspase levels did not differ from those in the Control (Figure 3A,B). As shown in the figure, EPA, CPA, and TPA in high-glucose medium decreased Bax and cleaved caspase levels compared to those in the HG medium (*p* < 0.05). Levels of Bcl-2 and caspase did not differ among all groups exposed to high-glucose medium, but the ubiquitinated protein levels were increased compared to those in the Control (*p* < 0.05; Figure 3A,B).

Ubiquitinated protein levels were significantly increased when exposed to high-glucose medium compared to the Control (*p* < 0.05; Figure 3C). PC12 cells exposed to high-glucose medium with EPA, CPA, or TPA showed decreased ubiquitinated protein levels compared to high-glucose medium (*p* < 0.05; Figure 3C).

#### 3.2.3. ROS Production and Apoptosis Induction in PC12 Cells

**Figure 4:**
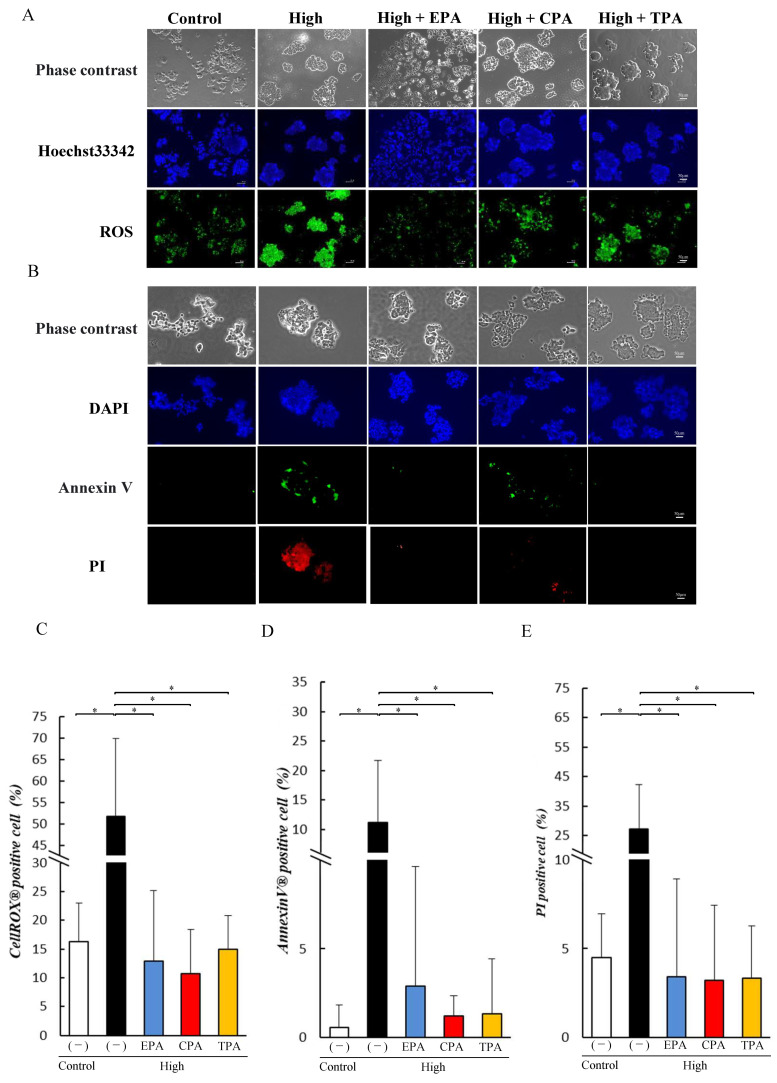
Effects of EPA, CPA, and TPA on intracellular reactive oxygen species (ROS) levels and apoptosis in PC12 cells exposed to high glucose. Control and high groups were prepared by culturing PC12 cells in 200 or 500 mg/dL glucose medium for seven days. PC12 cells were cultured in 500 mg/dL glucose medium supplemented with EPA, CPA, or TPA for seven days. PC12 cells were treated with 2.5 µM CellROX Green reagent for 15 min. Apoptotic PC12 cells were treated with annexin V-fluorescein isothiocyanate (FITC) and propidium iodide (PI) for 30 min. Each value represents the average (±SE) derived by counting 20 cells per group per experiment from three to five independent experiments. Quantification of the percentage of cells that stained positive for ROS (**A**). Percentage of ROS-positive cells (**B**). Quantification of the percentage of cells that stained positive for apoptosis (**C**). Percentage of annexin V-positive cells (**D**). Percentage of PI-positive cells (**E**). Each value represents the average (±SE). * *p* < 0.05 vs. Control. Scale bars: 50 µm.

Effects of unsaturated fatty acids on ROS production were analyzed by staining the cells with ROX Green (Figure 4A). The number of ROS positive cells increased in high glucose PC12 cells compared to the Control (Figure 4C), and PC12 cells in high-glucose medium supplemented with EPA, CPA, and TPA showed a significant decrease in ROS production compared to high glucose (*p* < 0.05; Figure 4C). We examined the effects of EPA, CPA, and TPA on the apoptosis of PC12 cells using Annexin V-FITC/PI double staining (Figure 4B). High increased the number of Annexin V and PI positive cells compared to Control (*p* < 0.05; Figure 4D,E) but PC12 cells cultured with EPA, CPA, and TPA was significantly reduced (*p* < 0.05) compared to the high group (Figure 4D,E).

### 3.3. Effects of Unsaturated Fatty Acids in Differentiated PC12 Cells Exposed to High-glucose medium

#### 3.3.1. AGEs, RAGE, and Insulin Signaling in Differentiated PC12 Cells

**Figure 5:**
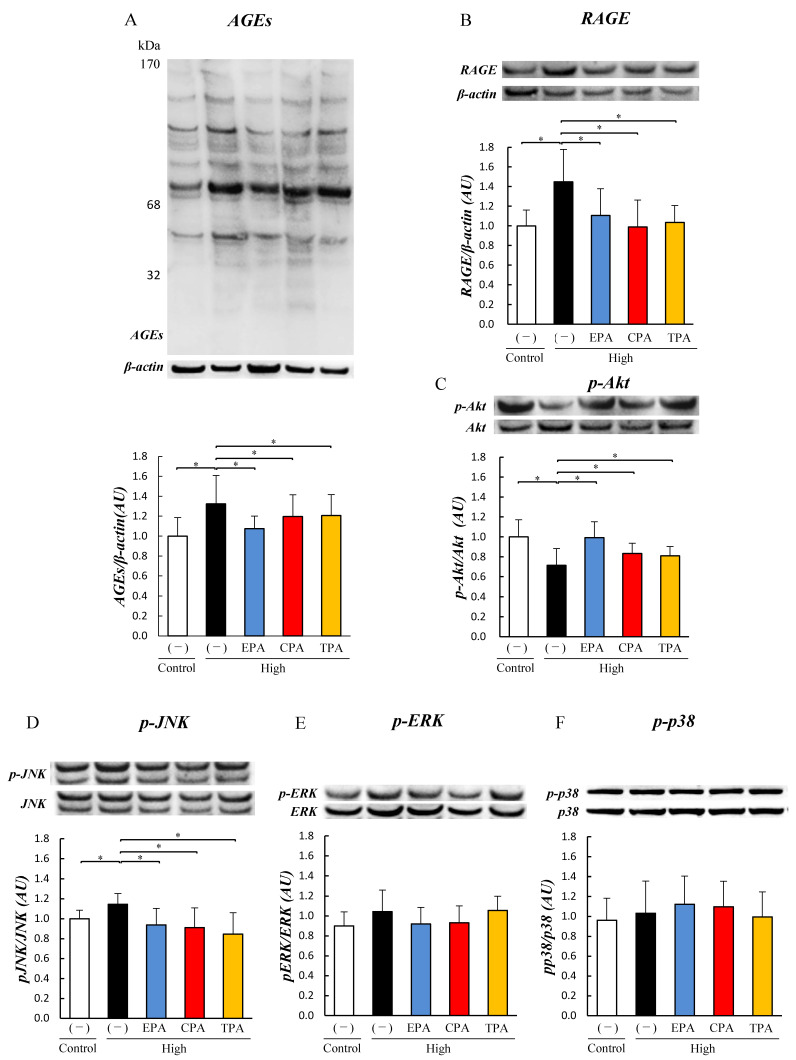
Effects of EPA, CPA, and TPA on the generation levels of AGEs and expression levels of RAGE in differentiated PC12 cells exposed to high glucose. Control and high groups were prepared by culturing PC12 cells in 200 or 500 mg/dL glucose medium for seven days. PC12 cells were cultured in 500 mg/dL glucose medium supplemented with EPA, CPA, or TPA for seven days. Differentiated cells were prepared by culturing the cells for seven days in a control medium supplemented with nerve growth factor (NGF) for two days. Levels of AGE, expression levels of RAGE, and levels of p-Akt, p-JNK, p-ERK, and p-p38 in differentiated PC12 cells were measured using Western blotting. Generation levels of AGEs (**A**). RAGE expression levels (**B**). Levels of p-Akt (**C**). p-JNK (**D**). p-ERK (**E**). and p-p38 (**F**). Each value represents the average (±SE). * *p* < 0.05 vs. Control.

Generation levels of AGEs and the expression of RAGE were higher in the high than in the Control but were greatly reduced in the high/EPA, high/CPA, and high/TPA (Figure 5A,B), and the level of p-Akt was significantly decreased in the high compared to the Control (*p* < 0.05; Figure 5C). When EPA, CPA, and TPA were added, the levels of p-Akt were significantly higher compared to high (*p* < 0.05) (Figure 5C). The level of p-JNK was not significantly different in high but showed a tendency to increase; the level of p-JNK in the high-glucose medium with EPA, CPA, and TPA also showed a tendency to decrease (*p* < 0.05) compared to the high, although not significantly different (Figure 5D) In differentiated PC12 cells, there was no significant difference in the levels of p-ERK and p-p38 between the Control, high, high/EPA, high/CPA, and high/TPA groups (Figure 5E,F).

#### 3.3.2. Apoptosis Signaling in Differentiated PC12 Cells

**Figure 6:**
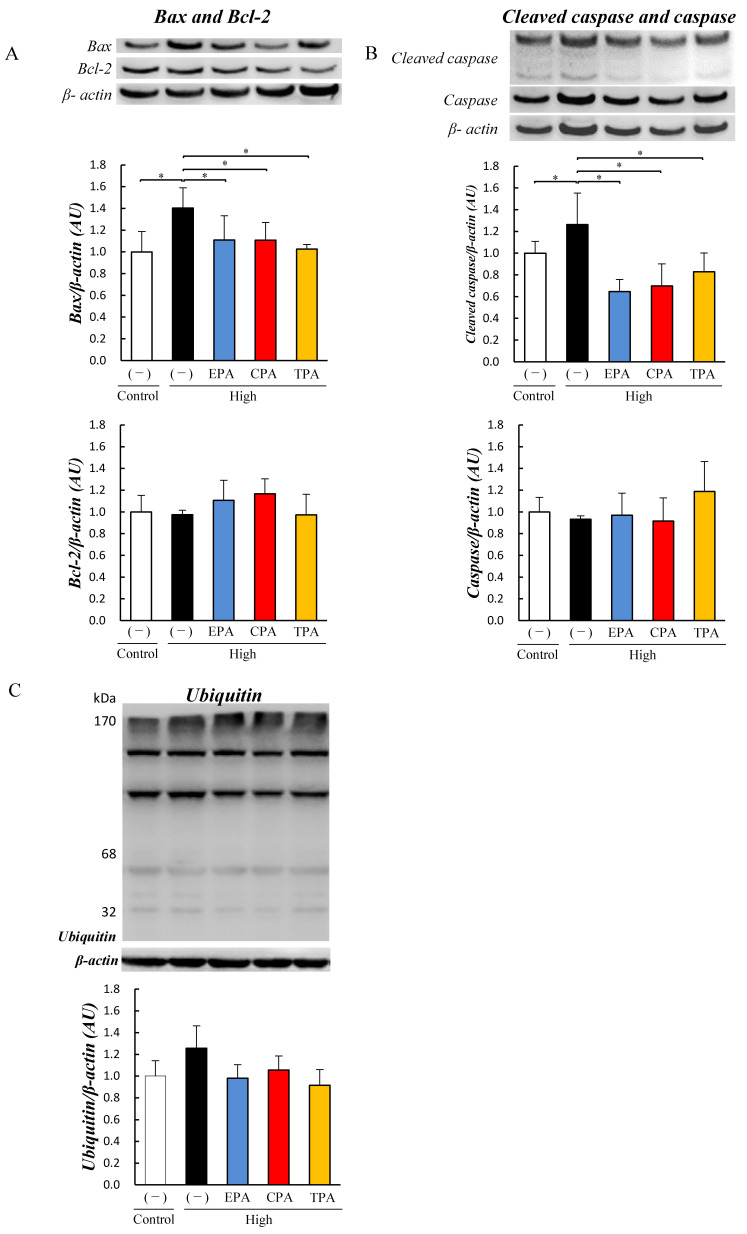
Effects of EPA, CPA, and TPA on apoptosis signaling expression in differentiated PC12 cells exposed to high glucose. Control and high groups were prepared by culturing PC12 cells in 200 or 500 mg/dL glucose medium for seven days. PC12 cells were cultured in 500 mg/dL glucose medium supplemented with EPA, CPA, or TPA for seven days. Differentiated cells were prepared by culturing the cells for seven days in a control medium supplemented with NGF for two days. Bax, Bcl-2, cleaved caspase, caspase, and ubiquitinated protein levels in differentiated PC12 cells were measured using Western blotting. Expression levels of Bax (**A**), Bcl-2 (**A**), cleaved caspase (**B**), caspase (**B**), and ubiquitinated protein (**C**). Each value represents the average (±SE). * *p* < 0.05 vs. Control.

In the high group, the expression of Bax and cleaved caspase protein was significantly higher than that in the Control, high/EPA, high/CPA, and high/TPA decreased the levels of Bax and cleaved caspase in high (*p* < 0.05; Figure 6A,B). There were no significant differences in the Bcl-2 and caspase levels among the Control, high, high/EPA, high/CPA, and high/TPA groups (Figure 6A,B). Ubiquitinated protein levels in differentiated PC12 cells were significantly increased when exposed to high-glucose medium compared to the Control (Figure 6C). Differentiated PC12 cells exposed to high-glucose medium with EPA, CPA, or TPA showed decreased ubiquitinated protein levels compared to high-glucose medium (*p* < 0.05; Figure 6C).

#### 3.3.3. ROS Production and Apoptosis Induction in Differentiated PC12 Cells

**Figure 7:**
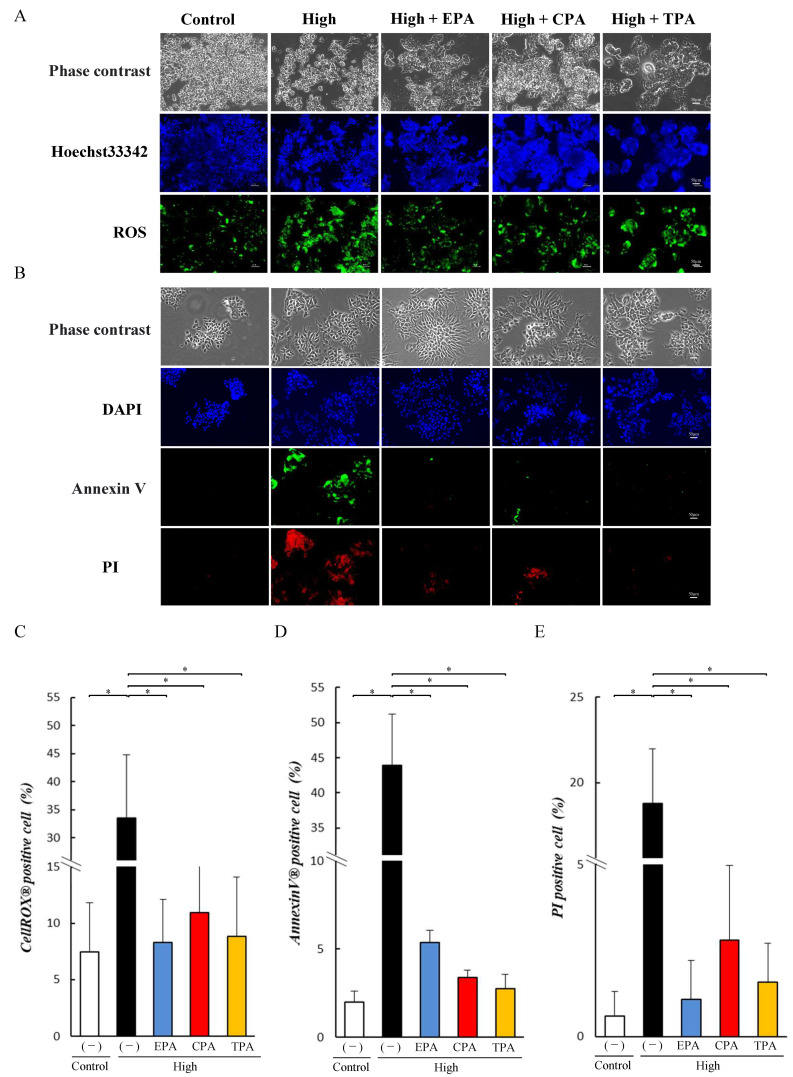
Effects of EPA, CPA, and TPA on intracellular ROS levels and apoptosis in differentiated PC12 cells exposed to high glucose. Control and high groups were prepared by culturing PC12 cells in 200 or 500 mg/dL glucose medium for seven days. PC12 cells were cultured in 500 mg/dL glucose medium supplemented with EPA, CPA, or TPA for seven days. Differentiated cells were prepared by culturing the cells for seven days in a control medium supplemented with NGF for two days. Differentiated PC12 cells were treated with 2.5 μM CellROX Green reagent for 15 min. Apoptotically differentiated PC12 cells were treated with annexin V-FITC and PI for 30 min. Each value represents the average (±SE) derived by counting 20 cells per group per experiment from 3–5 independent experiments. Each value represents the average (±SE). * *p* < 0.05 vs. Control. Quantification of the percentage of cells that stained positive for ROS (**A**). Percentage of ROS-positive cells (**C**). Quantification of the percentage of cells positive for apoptosis (**B**). Percentage of annexin V-positive cells (**D**). Percentage of PI-positive cells (**E**). Percentage of apoptosis-positive cells.

Number of ROS-positive cells was higher in the high compared to in Control and ROS levels were significantly lower in the high/EPA, high/CPA, and high/TPA groups than in high (*p* < 0.05; Figure 7A–E). The number of Annexin V and PI positive cells increased in high compared to the Control but significantly decreased in high/EPA, high/CPA, and high/TPA compared to high (*p* < 0.05; Figure 7A–E).

## 4. Discussion

Nutritional status of the fetus during the intrauterine period has a significant impact on long-term health. Malnutrition during the fetal period leads to diabetes and hypertension in adulthood [8,9,10,11]. However, children born from GDM or pre-gestational DM develop various complications due to exposure to intrauterine hyperglycemia during fetal life [17,18,19]. A hyperglycemic environment during pregnancy may affect fetal growth and differentiation; however, the underlying pathogenic mechanism is not well understood. In the present study, we analyzed the effects of hyperglycemia on the brain of rat pups at the molecular level using a GDM rat model that exhibits hyperglycemia during pregnancy. Previous studies using GDM rat models have reported increased neonatal stillbirth rates and an increased incidence of fetal malformations [37,38]. Piazza et al. also reported that hyperglycemia causes hippocampal developmental defects, neuronal apoptosis, decreased cell viability, and neuroinflammation in the brains of pups born to GDM rats [39]. Behavioral studies have reported increased anxiety-like behavior, decreased social interaction, and learning and memory deficits in female pups born to GDM model rats [40,41,42]. In contrast, serum and brain AGEs concentrations were significantly increased in STZ-induced mature diabetic models [43]. In the cerebrum of pups of the GDM rat model in this study, accelerated accumulation of AGEs and decreased phosphorylation levels of Akt (Figure 1) were observed. However, the administration of the n-7 unsaturated fatty acids CPA and TPA during pregnancy in GDM rat model inhibited the accumulation of AGEs and improved the inhibition of Akt phosphorylation in the cerebrum of pups (Figure 1). This suggests that maternal hyperglycemia during pregnancy affects the fetal brain and may be ameliorated using CPA and TPA. Therefore, we investigated the effects of a hyperglycemic environment during pregnancy on brain function in next-generation PC12 cells exposed to high glucose. PC12 cells have long been used as neuron-like model cells. However, they are limited by their inability to replicate the actual process of neuronal development.

AGEs that accumulate owing to prolonged exposure to hyperglycemia have been reported to be involved in the development of diabetic vascular complications [44,45,46,47]. AGEs are produced by non-enzymatic reactions between glucose and proteins and some AGEs are cytotoxic [48]. One of the molecular mechanisms leading to cytotoxicity is the induction of apoptosis via RAGE, a receptor for AGEs that promotes inflammatory signaling and ROS production and suppresses insulin signaling [49]. It has been reported that increased intracellular ROS cleaves effector caspases via Bax in mitochondria, activates apoptosis, and promotes the degradation of genomic DNA [50,51]. Recently, it has become clear that AGE-2, which is particularly toxic among AGEs, is widely involved not only in diabetic vascular complications, such as diabetic retinopathy and nephropathy caused by microangiopathy, but also in dementia and cancer [44,45,46,47,52]. PC12 cells, used as GDM model cells, were studied at 900–1350 mg/dL glucose in experiments by other groups. This high-glucose concentration (hyperglycemia) makes it difficult to continue pregnancy if replaced in humans. Therefore, in this experiment, we set the glucose concentration to 500 mg/dL and investigated its effect on cells. PC12 cells were exposed to a high-glucose medium containing 500 mg/dL glucose for 3 days, but there was no decrease in cell viability (Figure S1). First, to examine the effects of high glucose exposure on cells, we analyzed the expression of AGEs in proteins. The results showed that AGEs increased in PC12 cells exposed to a high-glucose medium (500 mg/dL), for 7 days, similar to the expression of RAGE, a receptor for AGEs (Figure 2). This is the first report on the expression levels of protein AGEs and RAGE in PC12 cells upon high-glucose exposure. The increase in AGE and RAGE levels in PC12 cells exposed to high-glucose medium was also consistent with the cerebrum of neonatal pups of GDM model animals (Figure 1), suggesting that exposure to high glucose levels increased protein AGE levels in the cerebrum of PDM and PC12 cells. We previously reported that hyperglycemic exposure during pregnancy inhibited insulin signaling in the hearts of GDM rat pups [23]. Therefore, we examined insulin signaling in the cerebrum of the PDM and PC12 cells. The results showed that Akt phosphorylation was inhibited in the PDM cerebrum and PC12 cells exposed to high-glucose medium. Lei et al. also reported that the exposure of PC12 cells to high glucose (900 mg/dL) reduced the phosphorylation levels of Akt and FOXO [53]. This suggests that insulin signaling is also inhibited in PC12 cells owing to increased AGE conversion caused by hyperglycemia.

Here, we investigated the effects of EPA, which has been reported to have anti-inflammatory effects, and CPA and TPA, which have been shown to improve AGE and Akt phosphorylation levels, in PC12 cells. Several epidemiological studies have reported that higher blood TPA levels are associated with higher insulin sensitivity and a lower incidence of type 2 diabetes [54,55,56,57]. The results of the current study showed that the addition of CPA or TPA to PC12 cells exposed to a high-glucose medium significantly decreased the levels of AGEs (Figure 2) and increased Akt phosphorylation (Figure 2). Chronic hyperglycemia has been shown to induce neurodegenerative diseases and diabetic retinopathy by inducing the production of inflammatory cytokines, reactive oxygen species, and oxidative stress [58]. It has also been shown that PC12 cells exposed to high glucose (1350 mg/dL) induce inflammation by increasing JNK phosphorylation due to increased intracellular ROS production [59]. In the present study, JNK phosphorylation of inflammatory signals was enhanced in PC12 cells exposed to high glucose levels (500 mg/dL; Figure 2), and ROS levels were increased (Figure 4). In contrast, the addition of TPA and CPA to PC12 cells exposed to high glucose decreased JNK phosphorylation (Figure 2) and ROS production (Figure 4), indicating that inflammation was suppressed. Oxidative stress (ROS) plays an important role in the development of various degenerative brain diseases, such as the Alzheimer’s and Parkinson’s diseases. Furthermore, chronic inflammation due to ROS overproduction is thought to be a major cause of cell damage and death [59]. Aminzadeh and Lei reported that apoptosis was induced in PC12 cells exposed to high glucose levels (1350 mg/dL by an increase in Bax, an apoptosis-promoting protein, and cleaved caspases [53,59]. Therefore, we examined whether apoptosis was induced in PC12 cells after exposure to high glucose. High glucose exposure activated Bax and increased the levels of cleaved caspases (Figure 3), suggesting that apoptosis was induced. FITC-labeled annexin V was used to confirm the binding of phosphatidylserine to the surface of the plasma membrane for exposure. These results suggested that exposure to a high glucose concentration (500 mg/dL) in PC12 induced apoptosis (Figure 4). This suggests that 7 days of high glucose exposure in PC12 cells activates pro-apoptotic proteins and induces apoptosis by activating inflammatory signalling pathways by ROS. Therefore, we examined the effects of adding fatty acids to the high-glucose medium and found a decrease in the expression of BAX and cleaved caspase (Figure 3) and a decrease in the number of apoptotic cells (Figure 4). This suggests that CPA and TPA may suppress inflammation by inhibiting the production of AGEs in the fetal cerebrum and may prevent apoptosis. However, continuous exposure of PC12 cells to CPA at a concentration of 300 µM for 72 h reduced cell viability (Figure S2). This indicated that prolonged exposure to CPA at 300 µM or higher may be toxic. Therefore, in the present experiment, 100 µM of CPA and TPA were replaced every 24 h in a high-glucose medium. TPA did not decrease cell viability at 300 µM, suggesting that the structural isomers CPA and TPA may have different effects on cells, even though they are palmitoleic acids. Therefore, TPA may be safer than CPA if administered orally by mothers. CPA is synthesized in the body from palmitic acid, whereas TPA is produced from trans-vaccenic acid, which is biosynthesized by bacteria in the gut of ruminants and is abundant in dairy products [30]. Furthermore, the blood TPA concentration is proportional to the amount of TPA-containing food consumed and is considered a biomarker for dairy products [29]. Consumption of dairy products containing TPA during pregnancy is expected to increase serum TPA concentrations. Therefore, based on the results of the present study, it is expected that the consumption of dairy products containing TPA will not only improve the glucose tolerance of the mother but also prevent the adverse effects of exposure to high blood glucose in the fetus.

**Figure 8:**
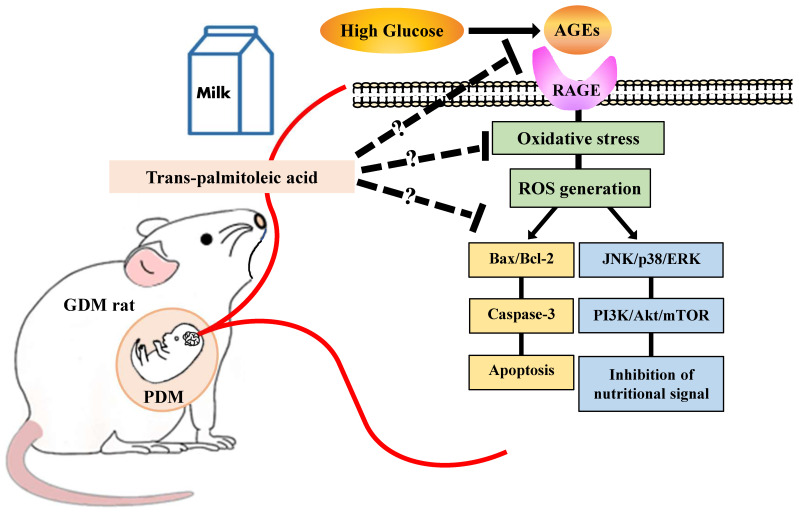
Palmitoleic acid ameliorates the effects of the hyperglycemic environment during pregnancy on the child. Exposure to a hyperglycemic environment during pregnancy leads to excessive protein glycation, inducing inflammation and apoptosis. However, these abnormalities can be ameliorated via palmitoleic acid intake during pregnancy. In the signaling pathways, the target molecule of palmitoleic acid has not been identified.

To study the effects on offspring after birth, the glucose concentration of the differentiation medium was set to that of the control medium (200 mg/dL glucose). PC12 cells exposed to a high-glucose medium for 7 days were differentiated by culturing in control medium supplemented with NGF for 48 h. The results showed that PC12 cells that differentiated in the control medium continued to be affected prior to differentiation. These results indicate that the hyperglycemic environment during pregnancy increases ROS and inflammatory signals via AGE–RAGE signaling and induces apoptosis (Figure 8). However, the present study revealed, for the first time, the possibility of avoiding this abnormality by consuming the n-7 unsaturated fatty acids, TPA and CPA, during pregnancy. As TPA in dairy products prevents programmed cell death in the cerebral neurons of pups born in a hyperglycemic environment during pregnancy, active consumption of dairy products by pregnant women may protect the fetal brain from neuronal degeneration in an intrauterine hyperglycemic environment.

Hyperglycemia is a serious condition caused by excessive sugar consumption. Considering the complexities associated with Alzheimer’s disease and diabetes, it is conceivable that exposure to a hyperglycemic intrauterine environment during fetal development can profoundly affect the differentiation and maturation of fetal cells and organs. In our study, we employed a rat model of intrauterine hyperglycemia to explore the significant effects of excessive glycation and oxidative stress on fetal organs.

Moreover, due to the limited availability of primary neurons for comprehensive biochemical analyses, we utilized PC12 cells, a widely used neuronal model in scientific research. Although the differentiation of PC12 cells using NGF allowed us to investigate pre- and post-differentiation effects, other factors, such as alterations in neuronal morphology and filopodia, could not be thoroughly explored. Therefore, to delve deeper into the effects of hyperglycemia and functional lipids on neuronal differentiation, including epigenetic analysis, it is imperative to conduct further investigations, especially in differentiating iPS cells into neurons.

Although humans have evolved mechanisms to mitigate oxidative stress, developing an anti-glycation system that can effectively mitigate the detrimental effects of excessive AGE formation, which leads to apoptosis, remains an unsolved matter. Building upon the findings of this study, our future goals include an extensive investigation into the molecular mechanisms through which functional lipids consumed by mothers, such as palmitoleic acid, effectively act as anti-glycation agents within the body. We firmly believe that these efforts will significantly contribute to improved fetal health.

## 5. Conclusions

In this study, we found that excessive protein glycation inhibited nutritional signaling in the cerebrum of pups born to a GDM rat model that mimicked the hyperglycemic environment during pregnancy via triggering inflammatory signaling. However, CPA and TPA suppressed protein glycation in the GDM rat model during pregnancy. Exposure of PC12 cells to a high-glucose medium for seven days enhanced the expression levels of AGEs and RAGE in GDM model cells, similar to animal studies in GDM model rats. These results suggest that AGEs induce ROS production and inflammatory signals, subsequently inducing apoptosis. Furthermore, the adverse effects of high glucose exposure on this cell model (inflammatory signaling, impaired insulin signaling, and apoptosis induction) persisted even after the cells continued to differentiate at normal glucose concentrations. However, TPA, an n-7 unsaturated fatty acid that does not exhibit cytotoxicity, inhibited AGE production and induced apoptosis in cells exposed to high glucose concentrations. These results suggest that for GDM fetuses, the hyperglycemic environment during pregnancy may cause programmed cell death of neurons; however, the intake of dairy products containing TPA, which the mother cannot synthesize in her body, may prevent these abnormalities and apoptosis due to AGE production that cause maternal and fetal complications. This study only used a cellular model with neural-like cells and did not reproduce actual fetal neurogenesis. We observed that the effects of exposure to a hyperglycemic environment continued after NGF differentiation. Further elucidation of these effects is necessary using epigenetic recordings in cellular models and behavioral analyses of children of animal models in future studies.

## Figures and Tables

**Table 1:** Body weights and blood glucose levels of the rat pup groups.

Parameter	PCM (*n* = 10)	PDM (*n* = 10)	PDM/CPA (*n* = 10)	PDM/TPA (*n* = 10)
Body weight (mean ± SE, g)	6.0 ± 0.4	6.1 ± 0.9	5.1 ± 0.5	6.2 ± 0.4
Blood glucose level (mean ± SE, mg/dL)	70.1 ± 13.0	72.3 ± 13.1	77.7 ± 25.0	70.0 ± 10.7

Each value represents the mean ± standard error (SE). The mean values in columns without a common letter are different, *p* < 0.05. PDM, rat pups of gestational diabetes mothers; PCM, rat pups of control mothers. Rat pups of gestational diabetes mother fed CPA or TPA were designated as PDM/CPA or PDM/TPA, respectively.

## Data Availability

All data generated or analyzed during this study are included in this published article and its supplementary information files.

## Supplementary Materials

The following supporting information can be downloaded at: https://www.mdpi.com/article/10.3390/nu15153434/s1, Figure S1: Effect of exposure of PC12 cells to high-glucose medium for 3–7 days, Figure S2: Effects of EPA, CPA, and TPA concentrations in control cells.
