# Supplementary figures and images for: Maternal n-7 Unsaturated Fatty Acids Protect the Fetal Brain from Neuronal Degeneration in an Intrauterine Hyperglycemic Animal Model
Source: Nutrients. 2023 Aug 3;15(15):3434. doi: 10.3390/nu15153434 (PMC10421171; doi:10.3390/nu15153434)

Supplemental Figure 2

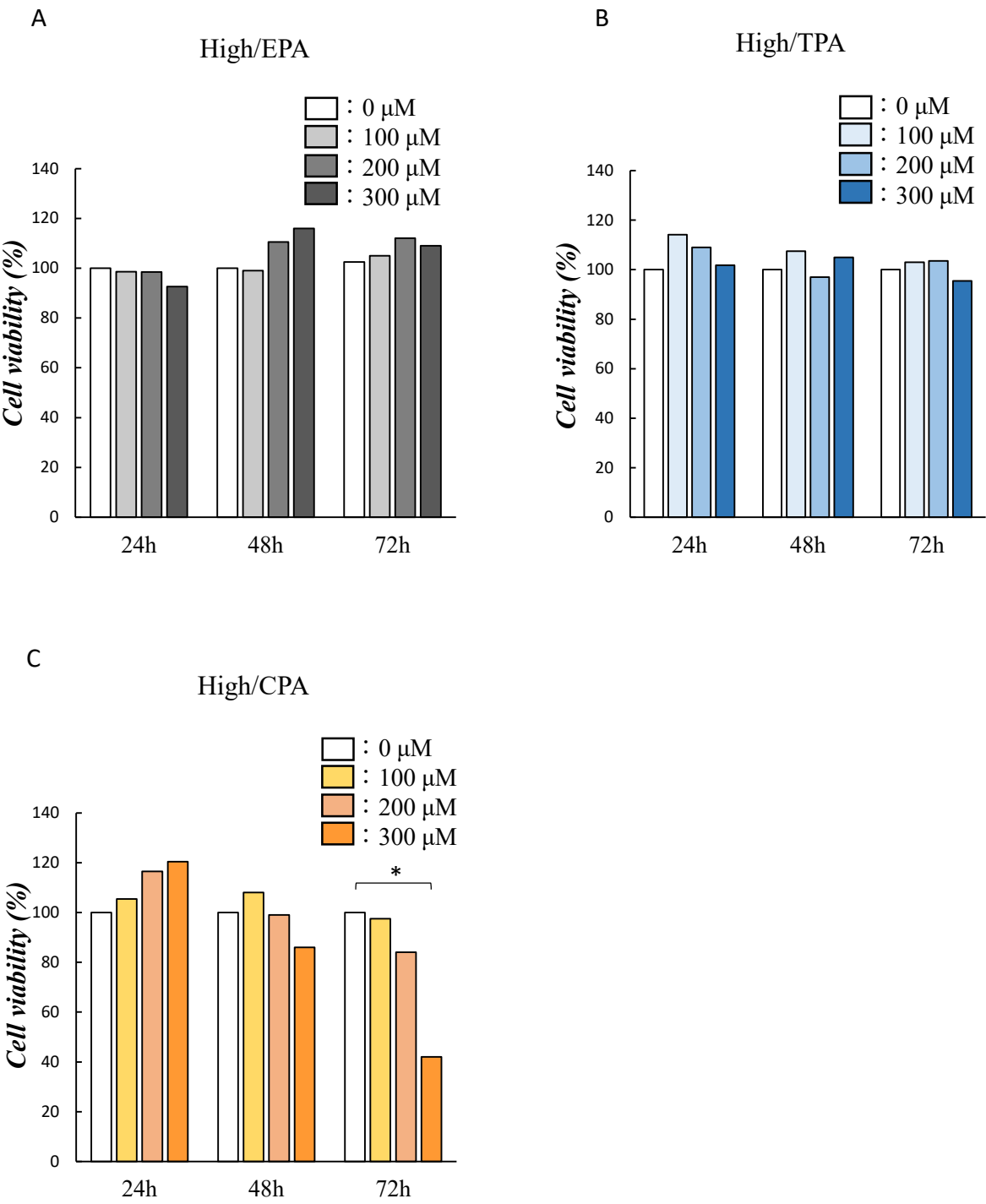

Supplement: Supplementary file 1 [file nutrients-15-03434-s001.zip › supplemental Figure02.pdf]

Supplemental Figure 1

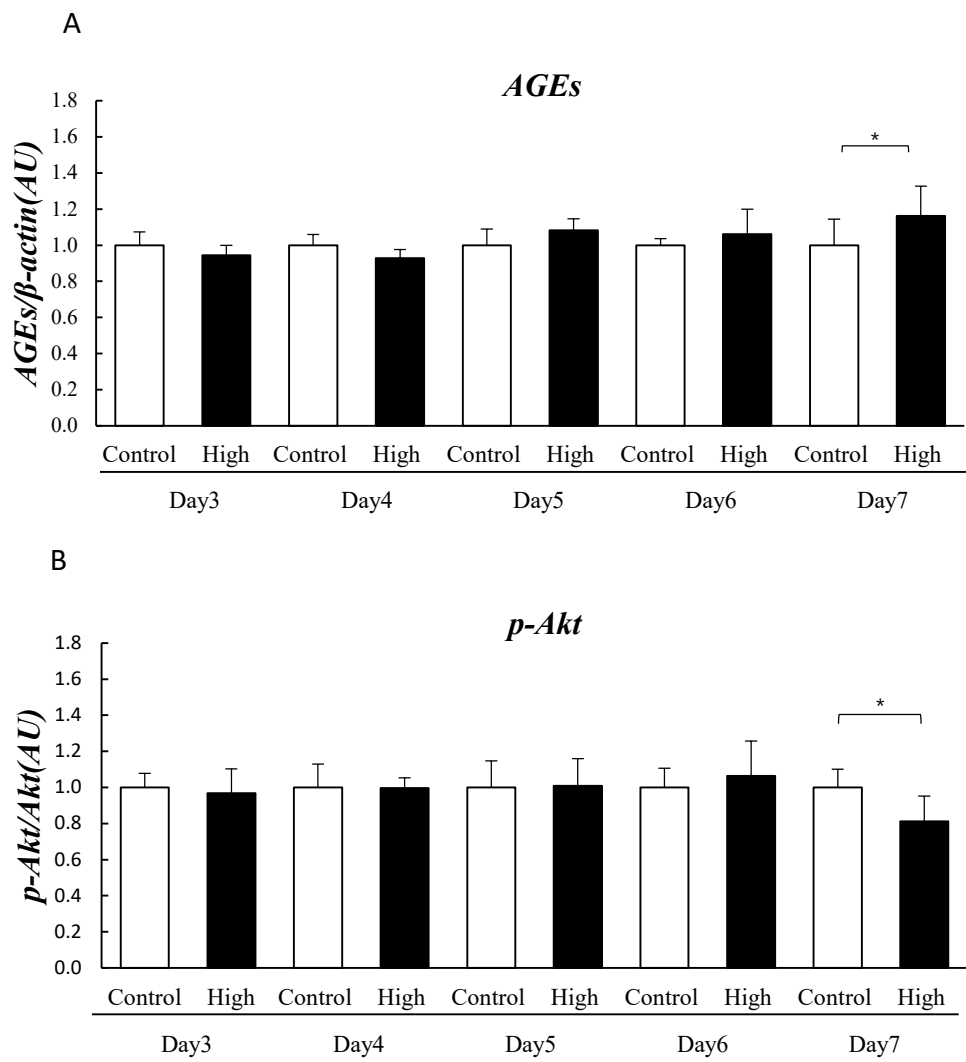

Supplement: Supplementary file 1 [file nutrients-15-03434-s001.zip › supplemental Figure01.pdf]
